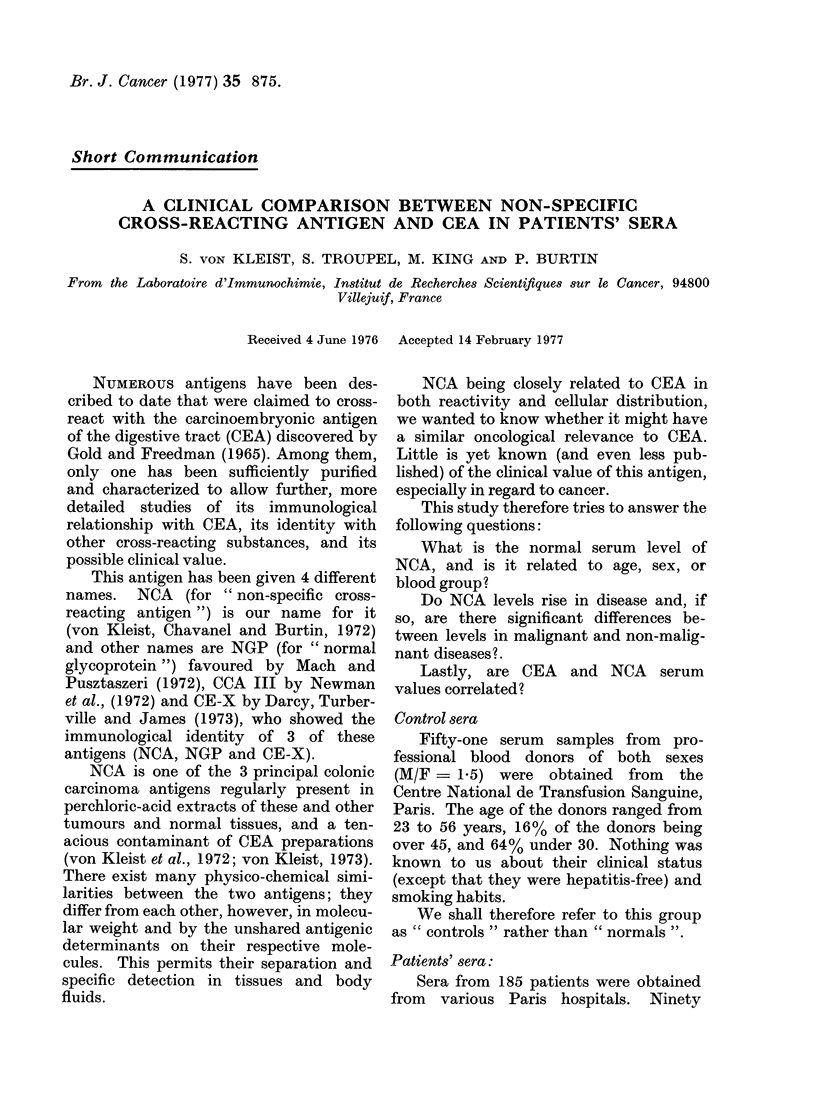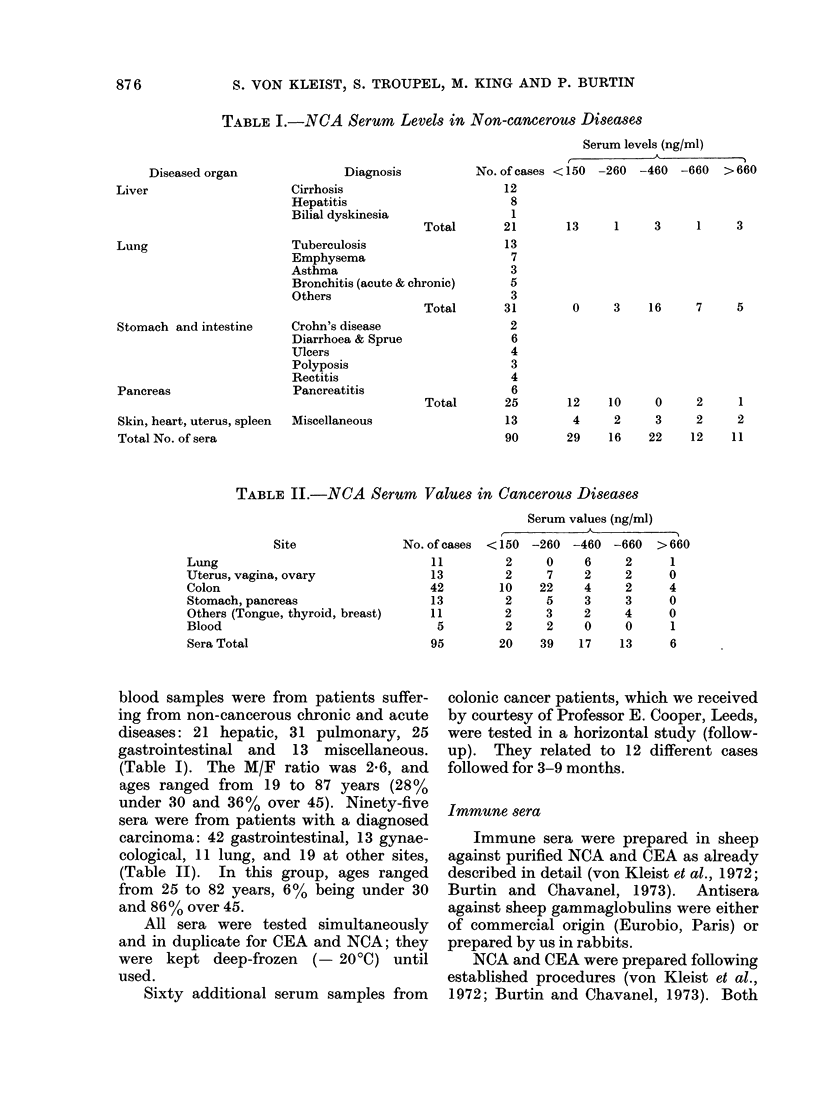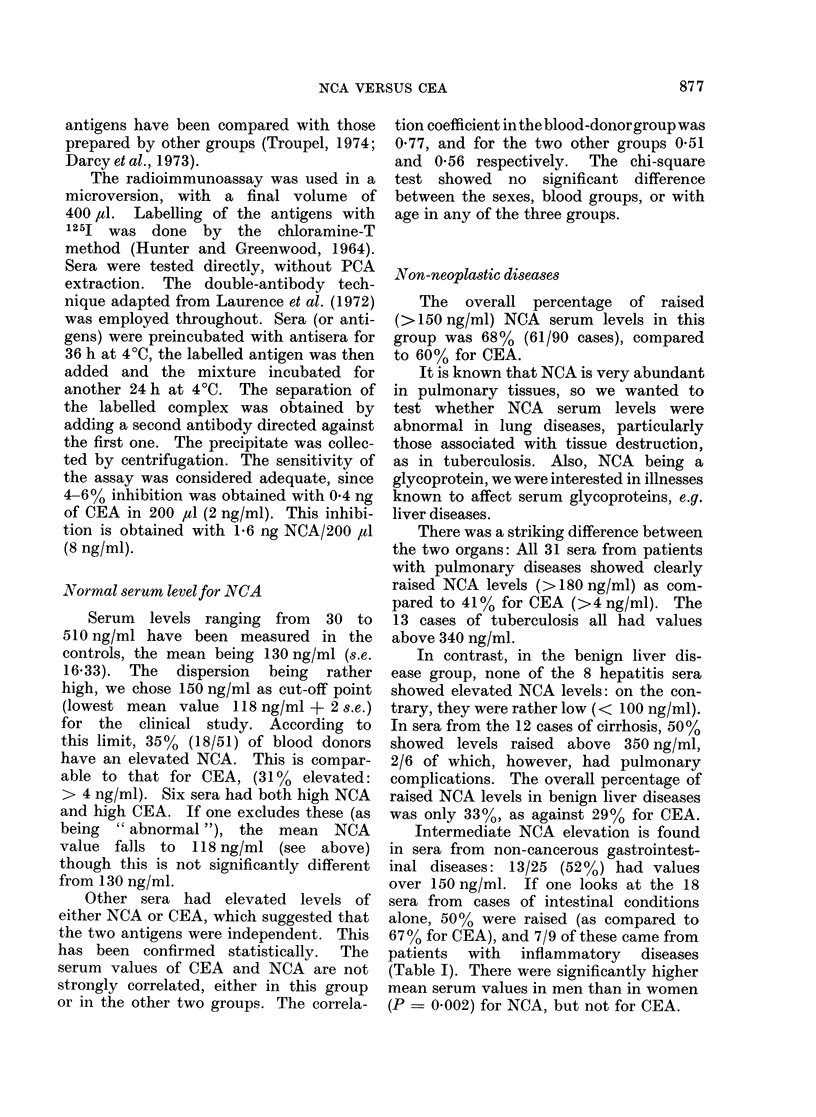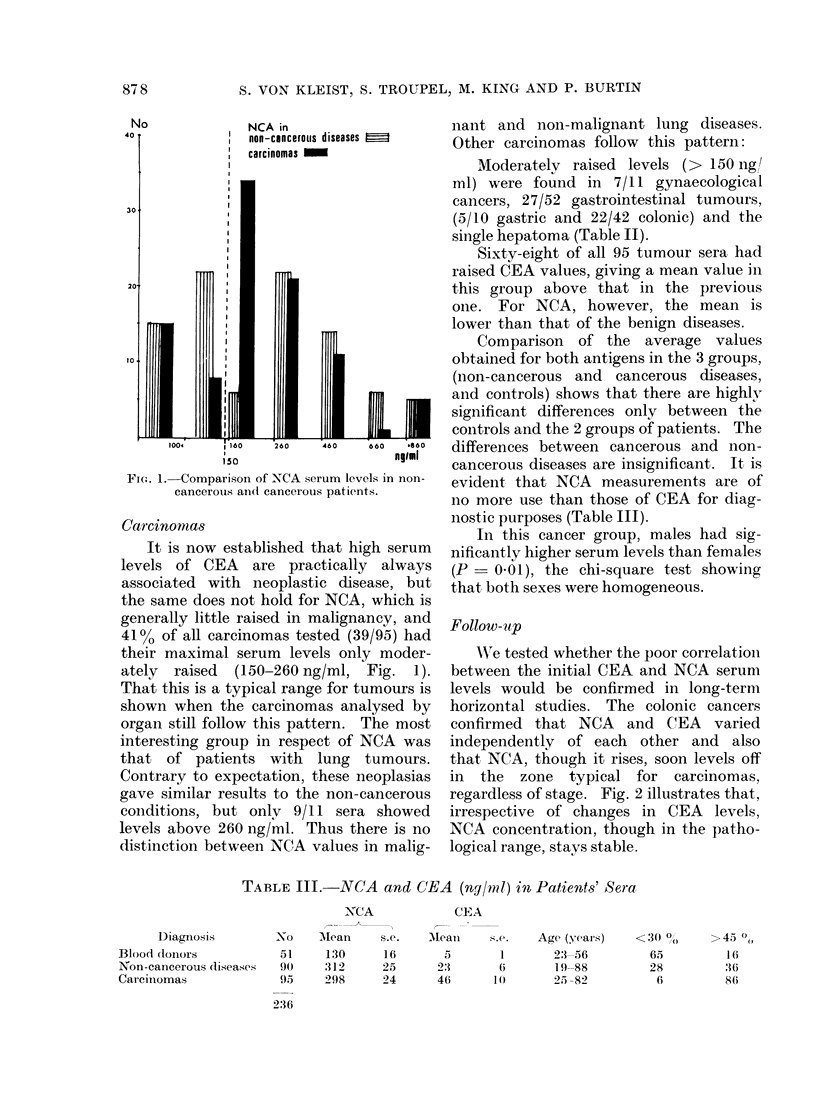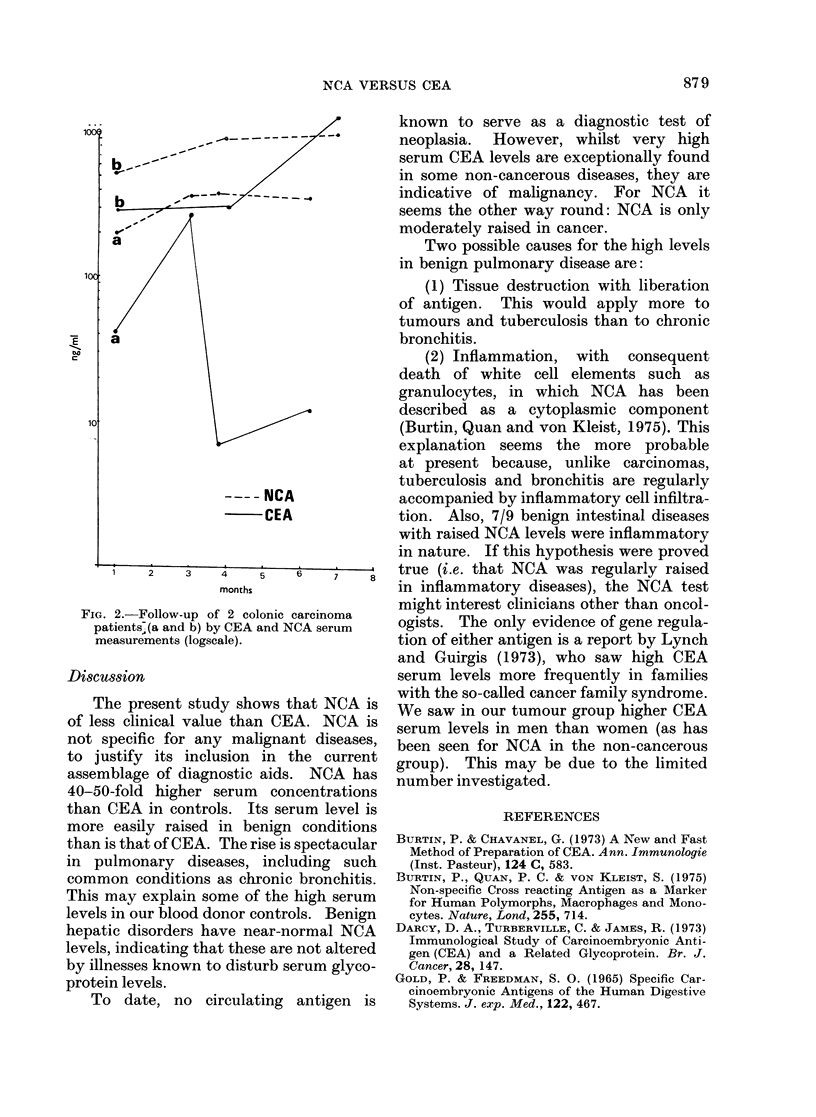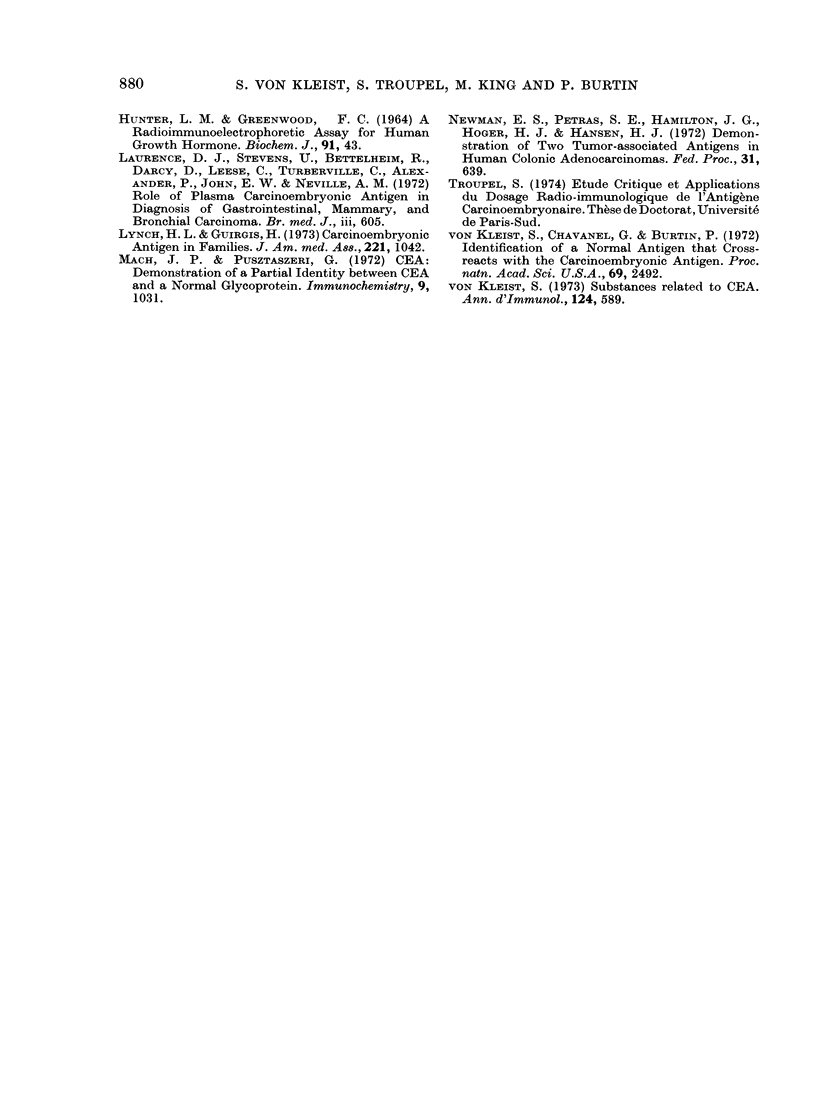# A clinical comparison between non-specific cross-reacting antigen and CEA in patient's sera.

**DOI:** 10.1038/bjc.1977.131

**Published:** 1977-06

**Authors:** S. von Kleist, S. Troupel, M. King, P. Burtin


*Br. J. Cancer* (1977) **35** 875.

***Short Communication***

### A CLINICAL COMPARISON BETWEEN NON-SPECIFIC CROSS-REACTING ANTIGEN AND CEA IN PATIENTS' SERA

S. VON KLEIST, S. TROUPEL, M. KING AND P. BURTIN

*From the Laboratoire d'Immunochimie, Institut de Recherches Scientifiques sur le Cancer, 94800 Villejuif, France*

Received 4 June 1976 Accepted 14 February 1977

NUMEROUS antigens have been described to date that were claimed to cross-react with the carcinoembryonic antigen of the digestive tract (CEA) discovered by Gold and Freedman (1965). Among them, only one has been sufficiently purified and characterized to allow further, more detailed studies of its immunological relationship with CEA, its identity with other cross-reacting substances, and its possible clinical value.

This antigen has been given 4 different names. NCA (for " non-specific cross-reacting antigen ") is our name for it (von Kleist, Chavanel and Burtin, 1972) and other names are NGP (for " normal glycoprotein ") favoured by Mach and Pusztaszeri (1972), CCA III by Newman *et al.*, (1972) and CE-X by Darcy, Turberville and James (1973), who showed the immunological identity of 3 of these antigens (NCA, NGP and CE-X).

NCA is one of the 3 principal colonic carcinoma antigens regularly present in perchloric-acid extracts of these and other tumours and normal tissues, and a tenacious contaminant of CEA preparations (von Kleist *et al.*, 1972; von Kleist, 1973). There exist many physico-chemical similarities between the two antigens; they differ from each other, however, in molecular weight and by the unshared antigenic determinants on their respective molecules. This permits their separation and specific detection in tissues and body fluids.

NCA being closely related to CEA in both reactivity and cellular distribution, we wanted to know whether it might have a similar oncological relevance to CEA. Little is yet known (and even less published) of the clinical value of this antigen, especially in regard to cancer.

This study therefore tries to answer the following questions:

What is the normal serum level of NCA, and is it related to age, sex, or blood group?

Do NCA levels rise in disease and, if so, are there significant differences between levels in malignant and non-malignant diseases?.

Lastly, are CEA and NCA serum values correlated?

*Control sera*

Fifty-one serum samples from professional blood donors of both sexes (M/F = 1·5) were obtained from the Centre National de Transfusion Sanguine, Paris. The age of the donors ranged from 23 to 56 years, 16% of the donors being over 45, and 64% under 30. Nothing was known to us about their clinical status (except that they were hepatitis-free) and smoking habits.

We shall therefore refer to this group as " controls " rather than " normals ".

*Patients' sera:*

Sera from 185 patients were obtained from various Paris hospitals. Ninety

TABLE I.—*NCA Serum Levels in Non-cancerous Diseases*

| Diseased organ | Diagnosis | No. of cases | Serum levels (ng/ml) <150 | −260 | −460 | −660 | >660 |
|---|---|---|---|---|---|---|---|
| Liver | Cirrhosis | 12 | | | | | |
| | Hepatitis | 8 | | | | | |
| | Bilial dyskinesia | 1 | | | | | |
| | Total | 21 | 13 | 1 | 3 | 1 | 3 |
| Lung | Tuberculosis | 13 | | | | | |
| | Emphysema | 7 | | | | | |
| | Asthma | 3 | | | | | |
| | Bronchitis (acute & chronic) | 5 | | | | | |
| | Others | 3 | | | | | |
| | Total | 31 | 0 | 3 | 16 | 7 | 5 |
| Stomach and intestine | Crohn's disease | 2 | | | | | |
| | Diarrhoea & Sprue | 6 | | | | | |
| | Ulcers | 4 | | | | | |
| | Polyposis | 3 | | | | | |
| | Rectitis | 4 | | | | | |
| Pancreas | Pancreatitis | 6 | | | | | |
| | Total | 25 | 12 | 10 | 0 | 2 | 1 |
| Skin, heart, uterus, spleen | Miscellaneous | 13 | 4 | 2 | 3 | 2 | 2 |
| Total No. of sera | | 90 | 29 | 16 | 22 | 12 | 11 |

TABLE II.—*NCA Serum Values in Cancerous Diseases*

| Site | No. of cases | Serum values (ng/ml) <150 | −260 | −460 | −660 | >660 |
|---|---|---|---|---|---|---|
| Lung | 11 | 2 | 0 | 6 | 2 | 1 |
| Uterus, vagina, ovary | 13 | 2 | 7 | 2 | 2 | 0 |
| Colon | 42 | 10 | 22 | 4 | 2 | 4 |
| Stomach, pancreas | 13 | 2 | 5 | 3 | 3 | 0 |
| Others (Tongue, thyroid, breast) | 11 | 2 | 3 | 2 | 4 | 0 |
| Blood | 5 | 2 | 2 | 0 | 0 | 1 |
| Sera Total | 95 | 20 | 39 | 17 | 13 | 6 |

blood samples were from patients suffering from non-cancerous chronic and acute diseases: 21 hepatic, 31 pulmonary, 25 gastrointestinal and 13 miscellaneous. (Table I). The M/F ratio was 2·6, and ages ranged from 19 to 87 years (28% under 30 and 36% over 45). Ninety-five sera were from patients with a diagnosed carcinoma: 42 gastrointestinal, 13 gynaecological, 11 lung, and 19 at other sites, (Table II). In this group, ages ranged from 25 to 82 years, 6% being under 30 and 86% over 45.

All sera were tested simultaneously and in duplicate for CEA and NCA; they were kept deep-frozen (— 20°C) until used.

Sixty additional serum samples from colonic cancer patients, which we received by courtesy of Professor E. Cooper, Leeds, were tested in a horizontal study (follow-up). They related to 12 different cases followed for 3–9 months.

*Immune sera*

Immune sera were prepared in sheep against purified NCA and CEA as already described in detail (von Kleist *et al.*, 1972; Burtin and Chavanel, 1973). Antisera against sheep gammaglobulins were either of commercial origin (Eurobio, Paris) or prepared by us in rabbits.

NCA and CEA were prepared following established procedures (von Kleist *et al.*, 1972; Burtin and Chavanel, 1973). Both antigens have been compared with those prepared by other groups (Troupel, 1974; Darcy *et al.*, 1973).

The radioimmunoassay was used in a microversion, with a final volume of 400 $\mu$l. Labelling of the antigens with $^{125}$I was done by the chloramine-T method (Hunter and Greenwood, 1964). Sera were tested directly, without PCA extraction. The double-antibody technique adapted from Laurence *et al.* (1972) was employed throughout. Sera (or antigens) were preincubated with antisera for 36 h at 4°C, the labelled antigen was then added and the mixture incubated for another 24 h at 4°C. The separation of the labelled complex was obtained by adding a second antibody directed against the first one. The precipitate was collected by centrifugation. The sensitivity of the assay was considered adequate, since 4–6% inhibition was obtained with 0·4 ng of CEA in 200 $\mu$l (2 ng/ml). This inhibition is obtained with 1·6 ng NCA/200 $\mu$l (8 ng/ml).

*Normal serum level for NCA*

Serum levels ranging from 30 to 510 ng/ml have been measured in the controls, the mean being 130 ng/ml (*s.e.* 16·33). The dispersion being rather high, we chose 150 ng/ml as cut-off point (lowest mean value 118 ng/ml + 2 *s.e.*) for the clinical study. According to this limit, 35% (18/51) of blood donors have an elevated NCA. This is comparable to that for CEA, (31% elevated: > 4 ng/ml). Six sera had both high NCA and high CEA. If one excludes these (as being "abnormal"), the mean NCA value falls to 118 ng/ml (see above) though this is not significantly different from 130 ng/ml.

Other sera had elevated levels of either NCA or CEA, which suggested that the two antigens were independent. This has been confirmed statistically. The serum values of CEA and NCA are not strongly correlated, either in this group or in the other two groups. The correlation coefficient in the blood-donor group was 0·77, and for the two other groups 0·51 and 0·56 respectively. The chi-square test showed no significant difference between the sexes, blood groups, or with age in any of the three groups.

*Non-neoplastic diseases*

The overall percentage of raised (>150 ng/ml) NCA serum levels in this group was 68% (61/90 cases), compared to 60% for CEA.

It is known that NCA is very abundant in pulmonary tissues, so we wanted to test whether NCA serum levels were abnormal in lung diseases, particularly those associated with tissue destruction, as in tuberculosis. Also, NCA being a glycoprotein, we were interested in illnesses known to affect serum glycoproteins, *e.g.* liver diseases.

There was a striking difference between the two organs: All 31 sera from patients with pulmonary diseases showed clearly raised NCA levels (>180 ng/ml) as compared to 41% for CEA (>4 ng/ml). The 13 cases of tuberculosis all had values above 340 ng/ml.

In contrast, in the benign liver disease group, none of the 8 hepatitis sera showed elevated NCA levels: on the contrary, they were rather low (< 100 ng/ml). In sera from the 12 cases of cirrhosis, 50% showed levels raised above 350 ng/ml, 2/6 of which, however, had pulmonary complications. The overall percentage of raised NCA levels in benign liver diseases was only 33%, as against 29% for CEA.

Intermediate NCA elevation is found in sera from non-cancerous gastrointestinal diseases: 13/25 (52%) had values over 150 ng/ml. If one looks at the 18 sera from cases of intestinal conditions alone, 50% were raised (as compared to 67% for CEA), and 7/9 of these came from patients with inflammatory diseases (Table I). There were significantly higher mean serum values in men than in women ($P = 0{\cdot}002$) for NCA, but not for CEA.

FIG. 1.—Comparison of NCA serum levels in non-cancerous and cancerous patients.

*Carcinomas*

It is now established that high serum levels of CEA are practically always associated with neoplastic disease, but the same does not hold for NCA, which is generally little raised in malignancy, and 41% of all carcinomas tested (39/95) had their maximal serum levels only moderately raised (150–260 ng/ml, Fig. 1). That this is a typical range for tumours is shown when the carcinomas analysed by organ still follow this pattern. The most interesting group in respect of NCA was that of patients with lung tumours. Contrary to expectation, these neoplasias gave similar results to the non-cancerous conditions, but only 9/11 sera showed levels above 260 ng/ml. Thus there is no distinction between NCA values in malignant and non-malignant lung diseases. Other carcinomas follow this pattern:

Moderately raised levels (> 150 ng/ml) were found in 7/11 gynaecological cancers, 27/52 gastrointestinal tumours, (5/10 gastric and 22/42 colonic) and the single hepatoma (Table II).

Sixty-eight of all 95 tumour sera had raised CEA values, giving a mean value in this group above that in the previous one. For NCA, however, the mean is lower than that of the benign diseases.

Comparison of the average values obtained for both antigens in the 3 groups, (non-cancerous and cancerous diseases, and controls) shows that there are highly significant differences only between the controls and the 2 groups of patients. The differences between cancerous and non-cancerous diseases are insignificant. It is evident that NCA measurements are of no more use than those of CEA for diagnostic purposes (Table III).

In this cancer group, males had significantly higher serum levels than females ($P = 0{\cdot}01$), the chi-square test showing that both sexes were homogeneous.

*Follow-up*

We tested whether the poor correlation between the initial CEA and NCA serum levels would be confirmed in long-term horizontal studies. The colonic cancers confirmed that NCA and CEA varied independently of each other and also that NCA, though it rises, soon levels off in the zone typical for carcinomas, regardless of stage. Fig. 2 illustrates that, irrespective of changes in CEA levels, NCA concentration, though in the pathological range, stays stable.

TABLE III.—*NCA and CEA (ng/ml) in Patients' Sera*

| | | NCA | | CEA | | | | |
|---|---|---|---|---|---|---|---|---|
| Diagnosis | No | Mean | s.e. | Mean | s.e. | Age (years) | <30 % | >45 % |
| Blood donors | 51 | 130 | 16 | 5 | 1 | 23–56 | 65 | 16 |
| Non-cancerous diseases | 90 | 312 | 25 | 23 | 6 | 19–88 | 28 | 36 |
| Carcinomas | 95 | 298 | 24 | 46 | 10 | 25–82 | 6 | 86 |
| | 236 | | | | | | | |

FIG. 2.—Follow-up of 2 colonic carcinoma patients (a and b) by CEA and NCA serum measurements (logscale).

### *Discussion*

The present study shows that NCA is of less clinical value than CEA. NCA is not specific for any malignant diseases, to justify its inclusion in the current assemblage of diagnostic aids. NCA has 40–50-fold higher serum concentrations than CEA in controls. Its serum level is more easily raised in benign conditions than is that of CEA. The rise is spectacular in pulmonary diseases, including such common conditions as chronic bronchitis. This may explain some of the high serum levels in our blood donor controls. Benign hepatic disorders have near-normal NCA levels, indicating that these are not altered by illnesses known to disturb serum glycoprotein levels.

To date, no circulating antigen is known to serve as a diagnostic test of neoplasia. However, whilst very high serum CEA levels are exceptionally found in some non-cancerous diseases, they are indicative of malignancy. For NCA it seems the other way round: NCA is only moderately raised in cancer.

Two possible causes for the high levels in benign pulmonary disease are:

(1) Tissue destruction with liberation of antigen. This would apply more to tumours and tuberculosis than to chronic bronchitis.

(2) Inflammation, with consequent death of white cell elements such as granulocytes, in which NCA has been described as a cytoplasmic component (Burtin, Quan and von Kleist, 1975). This explanation seems the more probable at present because, unlike carcinomas, tuberculosis and bronchitis are regularly accompanied by inflammatory cell infiltration. Also, 7/9 benign intestinal diseases with raised NCA levels were inflammatory in nature. If this hypothesis were proved true (*i.e.* that NCA was regularly raised in inflammatory diseases), the NCA test might interest clinicians other than oncologists. The only evidence of gene regulation of either antigen is a report by Lynch and Guirgis (1973), who saw high CEA serum levels more frequently in families with the so-called cancer family syndrome. We saw in our tumour group higher CEA serum levels in men than women (as has been seen for NCA in the non-cancerous group). This may be due to the limited number investigated.

### REFERENCES

BURTIN, P. & CHAVANEL, G. (1973) A New and Fast Method of Preparation of CEA. *Ann. Immunologie* (Inst. Pasteur), **124 C**, 583.

BURTIN, P., QUAN, P. C. & VON KLEIST, S. (1975) Non-specific Cross reacting Antigen as a Marker for Human Polymorphs, Macrophages and Monocytes. *Nature, Lond*, **255**, 714.

DARCY, D. A., TURBERVILLE, C. & JAMES, R. (1973) Immunological Study of Carcinoembryonic Antigen (CEA) and a Related Glycoprotein. *Br. J. Cancer*, **28**, 147.

GOLD, P. & FREEDMAN, S. O. (1965) Specific Carcinoembryonic Antigens of the Human Digestive Systems. *J. exp. Med.*, **122**, 467.

HUNTER, L. M. & GREENWOOD, F. C. (1964) A Radioimmunoelectrophoretic Assay for Human Growth Hormone. *Biochem. J.*, **91**, 43.

LAURENCE, D. J., STEVENS, U., BETTELHEIM, R., DARCY, D., LEESE, C., TURBERVILLE, C., ALEXANDER, P., JOHN, E. W. & NEVILLE, A. M. (1972) Role of Plasma Carcinoembryonic Antigen in Diagnosis of Gastrointestinal, Mammary, and Bronchial Carcinoma. *Br. med. J.*, iii, 605.

LYNCH, H. L. & GUIRGIS, H. (1973) Carcinoembryonic Antigen in Families. *J. Am. med. Ass.*, **221**, 1042.

MACH, J. P. & PUSZTASZERI, G. (1972) CEA: Demonstration of a Partial Identity between CEA and a Normal Glycoprotein. *Immunochemistry*, **9**, 1031.

NEWMAN, E. S., PETRAS, S. E., HAMILTON, J. G., HOGER, H. J. & HANSEN, H. J. (1972) Demonstration of Two Tumor-associated Antigens in Human Colonic Adenocarcinomas. *Fed. Proc.*, **31**, 639.

TROUPEL, S. (1974) Etude Critique et Applications du Dosage Radio-immunologique de l'Antigène Carcinoembryonaire. Thèse de Doctorat, Université de Paris-Sud.

VON KLEIST, S., CHAVANEL, G. & BURTIN, P. (1972) Identification of a Normal Antigen that Cross-reacts with the Carcinoembryonic Antigen. *Proc. natn. Acad. Sci. U.S.A.*, **69**, 2492.

VON KLEIST, S. (1973) Substances related to CEA. *Ann. d'Immunol.*, **124**, 589.